# Rising burden of severe pediatric coccidioidomycosis: a 25-year single-center study

**DOI:** 10.1093/jpids/piag019

**Published:** 2026-03-12

**Authors:** Sanchi Malhotra, Kristina Adachi, Ishminder Kaur, Paula Arribas Garcia, Paul Krogstad

**Affiliations:** Department of Pediatrics, Division of Infectious Diseases, David Geffen School of Medicine, University of California Los Angeles, Los Angeles CA, United States; Department of Pediatrics, Division of Infectious Diseases, David Geffen School of Medicine, University of California Los Angeles, Los Angeles CA, United States; Department of Pediatrics, Division of Infectious Diseases, David Geffen School of Medicine, University of California Los Angeles, Los Angeles CA, United States; Department of Pediatrics, Division of Infectious Diseases, David Geffen School of Medicine, University of California Los Angeles, Los Angeles CA, United States; Department of Pediatrics, Division of Infectious Diseases, David Geffen School of Medicine, University of California Los Angeles, Los Angeles CA, United States

**Keywords:** coccidioidomycosis, valley fever, pediatric infections, invasive fungal infections, antifungal agents, endemic mycoses

## Abstract

**Background:**

California’s incidence of pediatric coccidioidomycosis has risen considerably in the last 25 years, particularly in the last 3 years. Disseminated coccidioidomycosis is rare but associated with substantial morbidity. Sharing our recent and longitudinal pediatric experience can aid clinicians as the area of endemicity for this infection spreads.

**Methods:**

We performed a retrospective observational study of pediatric patients (0-17 years of age) with coccidioidomycosis evaluated at the University of California Los Angeles (UCLA) from January 1, 2000, to June 30, 2025.

**Results:**

Pediatric coccidioidomycosis cases at our institution increased significantly during the study period. One hundred thirty-four patients met our initial search criteria with 81 patients included in our final cohort. Of these, 72% (*n* = 58) had primary coccidioidomycosis and 28% (*n* = 23) had disseminated coccidioidomycosis, with 44% of the disseminated cases occurring between 2023 and 2025. Patients with disseminated disease had significantly longer hospitalizations [mean 144 days (95% CI, 81-207) vs 10 days (95% CI, 2-18), *P* < .001] and longer treatment durations [mean 26 months (95% CI, 9-42) vs 6 months (95% CI, 3-8), *P* < .001]. Patients with disseminated disease were significantly more likely to undergo therapeutic modification or additional therapy beyond fluconazole with 34% (*n* = 24) of all treated patients requiring a mold-active triazole as their final anti-fungal agent. Patients with disseminated disease were also significantly more likely than those with primary coccidioidomycosis to undergo surgical intervention as part of disease management [74% (*n* = 17) versus 26% (*n* = 6) *P* < .001].

**Conclusions:**

Our longitudinal experience as a regional referral center underscores the increasing clinical severity and resource burden of pediatric coccidioidomycosis in endemic areas, requiring multidisciplinary effort. This highlights the need for heightened clinical vigilance, earlier recognition of dissemination, advocating for subspecialty collaboration, and evaluation of optimal antifungal and adjuvant treatment strategies in children.

## INTRODUCTION

Coccidioidomycosis is a widely endemic fungal infection found in the southwestern United States and other areas in North, Central, and South America. While most cases in the United States are reported from California and Arizona, the known endemic area is expanding.^1^ Climate-based models incorporating temperature, precipitation, and incidence trends predict that the disease will be found throughout the western half of the continental United States by the year 2050.^2^ Correspondingly, the number of reported cases in endemic areas has risen. In California, the incidence of coccidioidomycosis among children and adolescents has dramatically increased from 1.8/100 000 in 2001 to 10.9/100 000 in 2024.^3^

Mid-20th-century coccidioidal skin test surveys in Southern California indicated that pediatric infections are substantially more common than the small proportion of patients who come to medical attention.^4^ Most infections are asymptomatic or mild, manifested only by low-grade fever, cough, and non-specific constitutional symptoms with eventual self-resolution. In contrast, infections accompanied by erythema nodosum (EN) or severe/progressive pulmonary symptoms are more likely to lead to diagnostic evaluation and confirmation of acute coccidioidomycosis.^5–7^ Although the true incidence of complicated disease in pediatrics is unknown, existing data suggest a substantial rate of dissemination among pediatric patients who present to care at regional centers, ranging from 17% to 42% in prior case series pre-2013.^8^^,^^9^

Published pediatric cohorts describe patients presenting prior to 2018; however, given the substantial increase in cases since then in California, updated data are needed.^8–10^ Therefore, we sought to provide a longitudinal review of patients at our center over the past 25 years. Our aim was to compare clinical features and management of patients with primary versus disseminated coccidioidomycosis, with particular attention to antifungal agent use amid increasing concerns about the efficacy of fluconazole and increased use of triazole mold-active agents.^11–14^

## METHODS

We conducted a single-center retrospective case review of pediatric patients diagnosed with coccidioidomycosis between January 1, 2000, and June 30, 2025, at the University of California Los Angeles (UCLA), including both inpatient and outpatient encounters.

We searched the electronic medical record (EMR) system for patients meeting the following criteria: patients aged 1 month to 17 years of age with ICD-9 codes 114.0-114.9 or ICD-10 codes B 38.0-B38.9, or a positive culture for *Coccidioides* spp., or a positive serum *Coccidioides* complement fixation titer. Molecular diagnostic methods such as nucleic acid–based detection were not used as part of search criteria as they are not yet validated for routine testing.^15^ Patients were excluded if they were ≥18 years at the time of diagnosis, were diagnosed prior to 2000, had the majority of their coccidioidomycosis management at another hospital, or lacked clinical documentation beyond the diagnosis code (Supplemental Figure 1). Patients were also excluded if they were deemed to have false-positive testing by the treating provider, most commonly an isolated positive IgM with negative immunodiffusion and complement fixation testing.^16^^,^^17^ Manual chart abstraction was performed to collect demographic characteristics, medical history, clinical presentations, laboratory and imaging findings, treatment regimens, and outcome data.

Patients were classified to have either (1) primary coccidioidomycosis, which encompassed patients who were asymptomatic or had localized pulmonary infection, or (2) disseminated coccidioidomycosis for patients with a site of extra-pulmonary disease. Patients were also subjected to categorization using a recently described clinicopathological schema to delineate features of complicated coccidioidomycosis.^18^

Statistical analysis included calculation of means with 95% confidence intervals and independent *t*-tests for continuous variables, and frequencies, odds ratios, chi-square analysis, and Fisher’s exact test for categorical variables. Statistics were performed using IBM SPSS Statistics Version 28.0 (IBM Corp, Armonk, NY). This study was approved by the institutional review board at the UCLA.

## RESULTS

### Demographics

**Figure 1:**
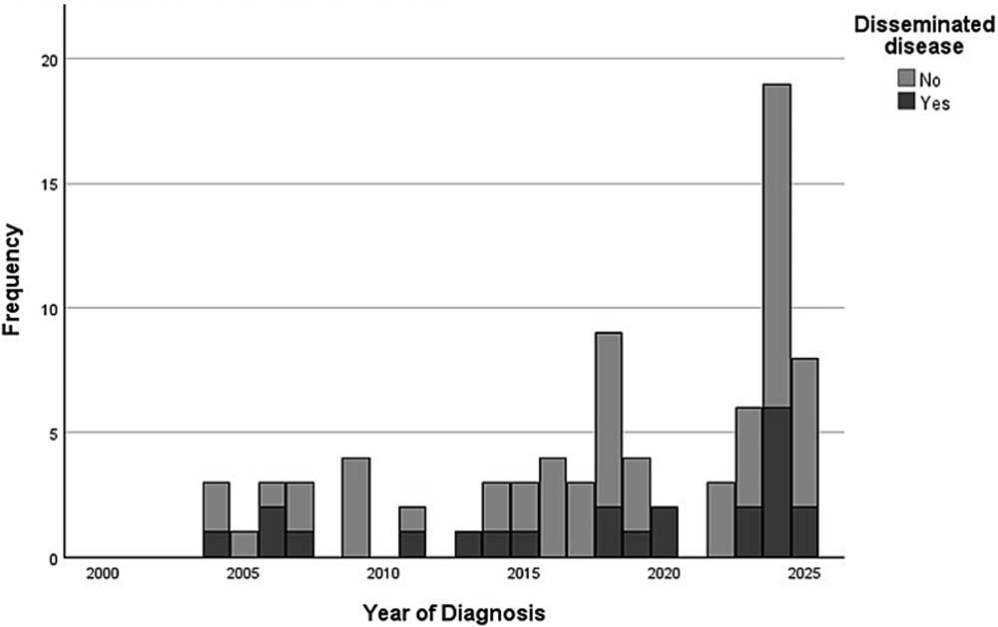
Cases of pediatric coccidioidomycosis seen at the University of California Los Angeles by year and disease type. Cases of coccidioidomycosis trended upward at this institution over time, with notable increases in 2018 and 2024. Please note that only the first 6 months of data are included in 2025

A total of 134 patients met initial EMR search criteria, of whom 81 met inclusion criteria after chart review (Supplemental Figure 1). Of the 81 patients, 72% (58) had primary coccidioidomycosis and 28% (23) had disseminated coccidioidomycosis. The number of patients evaluated at our center increased from 2000 to 2025, with large increases in patients diagnosed during 2018 and 2024 (Figure 1). Our data corresponded with increasing incidence during this time across the state.^3^

We conducted analyses to compare patients with primary versus disseminated coccidioidomycosis. Patients with disseminated coccidioidomycosis had an older mean age. Notably, 4 of 5 patients of Black or African American heritage had disseminated disease. There was no difference in dissemination based on immunocompromised status. Patients with dissemination were much more likely to require an inpatient stay and had significantly longer hospitalizations (Table 1). Many of the patients resided in Kern County (40.7%), followed by Los Angeles (18.5%) and Ventura County (8.6%).

**Table 1:** Demographic data of pediatric patients with coccidioidomycosis, comparing primary versus disseminated disease

	All patients (*n* = 81)	Primary coccidioidomycosis (72%, *n* = 58)	Disseminated coccidioidomycosis (28%, *n* = 23)	*P*-value
Age (mean)	11.8 (95% CI, 10.8-12.9)	12.5 (95% CI, 11.3-13.6)	10.2 (95% CI, 7.9-12.6)	*P* = .046
Sex (male)	56% (45)	57% (33)	52% (12)	*P* = .7
Race				
White	33% (27)	36% (21)	26% (6)	
Black or AA	6% (5)	2% (1)	17% (4)	
Asian/NH/PI	6% (5)	5% (3)	4% (2)	
American Indian	1% (1)	2% (1)	0	
Other	16% (13)	17% (10)	13% (3)	
Unknown	37% (30)	40% (22)	35% (8)	
Ethnicity (Hispanic/Latino/a/x)	40% (25)	39% (17)	40% (8)	*P* = .92
Immunocompromised (Y)	9% (7)	7% (4)	13% (3)	*P* = .4
Solid organ transplant	2% (2)	2% (1)	4% (1)	
Malignancy	2% (2)	2% (1)	4% (1)	
Autoimmune disease	2% (2)	3% (2)	0	
Innate immunodeficiency	1% (1)	0	4% (1)	
Insurance				
Medicaid	38% (31)	33% (19)	52% (12)	*P* = .13^a^
Commercial	41% (33)	45% (26)	30% (7)	
Other	4% (3)	5% (3)	0	
Unknown	17% (14)	17% (10)	17% (4)	
Hospitalization^b^	59% (47)	47% (27)	87% (20)	*P* = .001
Length of stay (mean)^c^	72 (95% CI 36.7-107.2)	9.9 (95% CI 1.8 – 17.9)	143.8 (95% CI 80.6-207.1)	*P* < .001

a Comparing Medicaid vs commercial only.

b Data missing for 1 patient.

c Data only available for total 41 patients (22 pulmonary, 19 disseminated).

### Clinical Symptoms

There was no difference in duration of symptoms prior to diagnosis between those with primary versus disseminated coccidioidomycosis (78 vs 79 days *P* = .97). Patients with primary disease were significantly more likely to present with cough and chest pain compared to those with disseminated disease. They also trended toward presenting with erythema nodosum and shortness of breath more frequently; however, this was not statistically significant due to the small number of cases in the disseminated cohort. Patients with disseminated disease were more likely to present with CNS symptoms, skin lesions, or cervical lymphadenopathy (Table 2).

**Table 2:** Symptoms reported at onset of illness comparing pediatric patients with primary versus disseminated coccidioidomycosis

Symptoms	Total % (N = 81)	Primary Coccidioidomycosis (N = 58)	Disseminated Coccidioidomycosis (N = 23)	OR (95% CI)	*p*-value^*^
Fever	68% (55)	71% (41)	61% (14)	1.6 (0.6-4.2)	0.4
Cough	48% (39)	57% (33)	26% (6)	3.7 (1.3-10.9)	0.012
Fatigue	28% (23)	35% (20)	13% (3)	3.5 (0.93-13.3)	0.061
Chest Pain	28% (23)	40% (22)	4% (1)	13.4 (1.7-107)	0.002
Musculoskeletal Pain	25% (20)	22% (13)	30% (7)	0.66 (0.2-2.0)	0.45
SOB/Resp failure	12% (10)	16% (9)	4% (1)	4.0 (0.5-33.9)	0.27
Flu-like symptoms^a^	20% (16)	19% (11)	22% (5)	0.8 (0.3-2.8)	0.77
Erythema Nodosum	19% (15)	24% (14)	4% (1)	7.0 (0.9-56.7)	0.055
Rash (EM, maculopapular)	14% (11)	17% (10)	4% (1)	4.6 (0.6-38.0)	0.17
CNS Symptoms (HA, seizure)^b^	16% (13)	10% (6)	30% (7)	0.3 (0.08-0.9)	0.026
Constitutional Symptoms (weight loss, night sweats)	17% (14)	16% (9)	22% (5)	0.7 (0.2-2.2)	0.53
Skin lesions	7% (6)	2% (1)	22% (5)	0.06 (.007-.6)	0.006
Cervical LAD/neck mass	4% (3)	0	13% (3)		0.021

Abbreviations: CNS, central nervous system; EM, erythema multiforme; HA, headache; LAD, lymphadenopathy; SOB, shortness of breath.

a Flu-like symptoms include malaise, myalgias, rhinorrhea, sore throat, and congestion.

b One patient with meningitis had seizure, and 1 patient with meningitis had photo/phonophobia; the rest of the patients in this row had headaches.

### Laboratory Diagnosis

*Coccidioides* EIA testing was the most sensitive among our patients, with 97% positivity in the 69 patients (84%) who had a test available in our EMR. Immunodiffusion testing was available in 80% (*n* = 65) of cases with 92% positivity. All 81 of our patients had a serum complement fixation titer performed around the time of diagnosis, with 91% positivity. Patients with disseminated disease were significantly more likely to have a complement fixation titer >1:16 near the time of diagnosis (56% vs 30%, *P* = .02). Nineteen patients in our cohort had *Coccidioides* identified either via pathology or culture, and 10 patients had susceptibility data available, largely (6/10) from 2024. Isolates from 5 (50%) of our patients had elevated fluconazole minimum inhibitory concentration (MIC) *>* 16, with 80% of those in 2024-2025, and all fluconazole MICs in this cohort were *>*8.^13^ The MICs for mold-active azoles remained low, all *<*1.

### Clinical Variants

We also categorized patients according to a recently described clinicopathological classification system to provide a detailed characterization of the manner of presentation and severity (Supplemental Table 1).^18^

**Figure 2:**
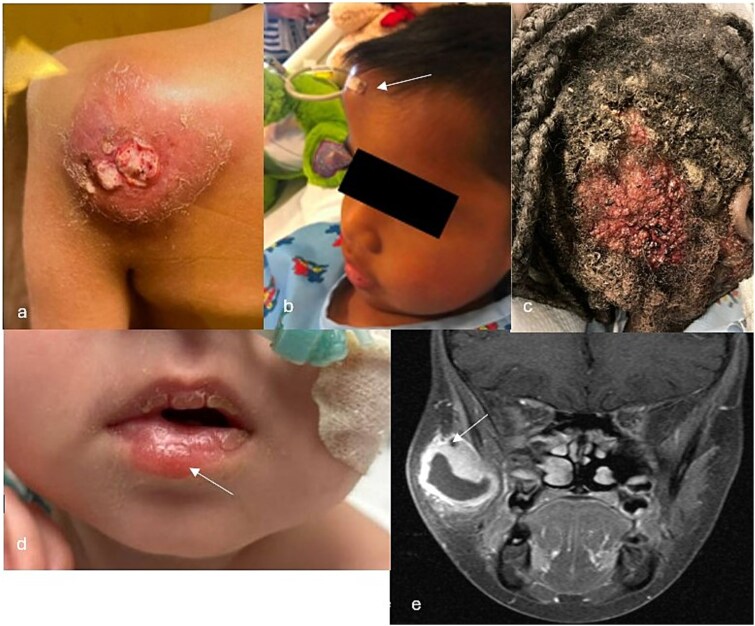
Photo collage of interesting clinical presentations, these represent patient sites of infection that all grew *Coccidioides* in culture obtained during biopsy or debridement. (a) Ulcerated mass on the supraspinatus muscle with scapular involvement. (b) Right frontal bone protruding lesion (MRI previously published).^19^ (c) Parietooccipital scalp lesion that invaded underlying bone (previously published).^20^ (d) Cutaneous lip nodule. (e) Right masseter muscle abscess with bony destruction of the right zygomatic arch

Most patients had uncomplicated pulmonary disease, with only 4 patients with cavitary disease, and 2 with respiratory failure from *Coccidioides*. Of patients with dissemination, 13 (57%) had CNS involvement, and 11 (48%) had musculoskeletal (MSK) disease. Patients with skin and MSK disease often had unique presentations that delayed diagnosis, as shown in Figure 2.

Of 23 (28%) patients requiring surgical intervention, 8 underwent bony/abscess debridement, 7 required a ventricular shunt or reservoir, 4 required a chest tube, 3 required a lung biopsy (2 diagnostic, 1 diagnostic and therapeutic with wedge resection), and 1 cervical node excision due to a fistula. Five patients with CNS disease received IT amphotericin (described further in a prior publication).^21^ One underwent craniotomy and removal of a dural-based lesion. Surgical intervention was significantly more common in patients with disseminated disease (74%, *n* = 17) compared to patients with primary disease (26%, *n* = 6), *P* < .001.

### Treatment

In this cohort, 88% (71) of patients were initiated on antifungal therapy, of whom 41% (29) required an additional medication or a change in medication per their infectious disease provider. Patients with dissemination were significantly more likely to require multiple medications [83% versus 21%; OR 21 (95% CI, 6-77) *P* < .001] than those with primary disease, and more likely to receive an amphotericin B formulation at some point during their treatment course (OR 10.6; 95% CI, 4.1-27.5, *P* < .001).

Of the 71 patients initiated on antifungal therapy, 90% initially received fluconazole, but only 65% remained on fluconazole as final therapy (only 35% of those with dissemination). Among all treated patients, 16% (11), 13% (9), 3% (2), and 3% (2) were eventually transitioned to posaconazole, isavuconazole, voriconazole, or itraconazole, respectively, as final therapy. Two patients also received concurrent olorofim, a novel first-in-class antifungal, on a compassionate use basis. Of patients with dissemination, 65% (15) required a change in medication compared to 21% (10) of those with primary coccidioidomycosis who received treatment. Only 1 of 13 patients with coccidioidal meningitis (CM) was managed on fluconazole alone during their course, 12 (92%) received concurrent liposomal amphotericin B for concurrent severe pulmonary or non-CNS extrapulmonary disease, and 10 (77%) eventually received a different azole. In addition, 11 patients received concurrent IFN gamma (Actimmune), and 6 patients received dupilumab per our immunology colleagues.

### Adverse Effects of Antifungal Therapy

Adverse effects commonly occurred among the 22 patients receiving a formulation of intravenous amphotericin, largely liposomal amphotericin B, except 1 patient who received amphotericin B lipid complex (ABLC) and 2 patients who received amphotericin B deoxycholate with 1 transitioning to ABLC. Of these 22 patients, 59% (13) experienced either electrolyte disturbances or acute kidney injury, with no lasting effects. Adverse effects with azole therapy were uncommon overall but most often encountered with fluconazole, which caused alopecia (4), GI upset (4), liver injury, and prolonged QT and torsades de pointes in combination with other medications in 1 patient. One patient on voriconazole developed visual disturbances, and 1 developed voriconazole-induced fluorosis. Posaconazole and isavuconazole were well tolerated without documented adverse effects.

### Pharmacologic Considerations

Therapeutic drug monitoring was always performed when mold-active triazoles were used, and less often (17%, *n* = 11) for patients receiving fluconazole. Serum drug concentrations were routinely obtained when itraconazole and voriconazole were prescribed but, due to the small sample size, were not further analyzed. The dosing regimens used yielded similar maximum troughs with posaconazole (mean: 5.0 mcg/mL, range 1.8-9.5, *n* = 9) and isavuconazole (mean: 4.7 mcg/mL, range 3.1-7.6, *n* = 11) (*P* = .77). There was also no significant difference in the number of days it took to achieve goal concentrations (typically trough concentrations of >2-3 mcg/mL)^15^^,^^22^^,^^23^ between posaconazole (Mean 21 days range 5-58) and isavuconazole (mean 18 days range 7-49) (*P* = .74). Higher doses of posaconazole up to 600 mg/day were well tolerated. Similarly, high doses of isavuconazole up to 22 and 36 mg/kg/day were needed in 2 patients to achieve desired troughs above 3.0 mcg/mL (per local expert opinion), with both testing negative for altered metabolism and neither experiencing adverse effects.

### Outcomes

We analyzed antifungal treatment duration for 47 patients (excluding those not on treatment, duration unable to be calculated due to lost follow-up, on lifelong treatment, or still amidst an ongoing treatment course) and found patients with dissemination received treatment for an average of 20 months longer than those with primary coccidioidomycosis (26 months vs 6 months, *P* < .001). No patients with dissemination were lost to follow-up compared to 26% of those with primary coccidioidomycosis (*P* = .006). Three deaths in our cohort were attributable to coccidioidomycosis, 2 from meningitis, and 1 from pulmonary disease with acute respiratory distress syndrome (ARDS). Excluding patients who were lost to follow-up (15) or deceased (3), 90% (39) of patients with primary coccidioidomycosis had resolved infection and completed treatment compared to only 26% (6) of patients with disseminated disease (*P* < .001).

## DISCUSSION

With climate change and increasing swings between rainy and drought periods in California, the population and pediatric incidence of coccidioidomycosis have increased substantially in the last 3 years. It is predicted to remain at historical highs in endemic areas while expanding its geographic area of endemicity.^1^^,^^24^^,^^25^ This 25-year retrospective review demonstrates both an increase in the total number of patients referred to our center and an increase in the complexity of their illnesses. Only 6 months of data were collected in 2025, but already more patients had presented than in all other years except 2018 and 2024. Notably, 44% of patients with disseminated coccidioidomycosis were seen in the last 2.5 years. Prior pediatric series also describe the varied presentations associated with this infection in California^8–10^^,^^26^; however, this is the most up-to-date series including treatment in an era of easier access to mold-active azoles, novel antifungals, and new immunologic therapies. Such data may help clinicians in emerging endemic areas anticipate unique presentations, management strategies, and the breadth of subspecialty support required.

Presenting symptoms were as expected, with fever, cough, chest pain, and constitutional symptoms being common, particularly among patients with primary coccidioidomycosis. Interestingly, while erythema nodosum is thought to indicate the presence of protective cellular immune responses, we encountered 1 patient who developed disseminated disease 6 months after the resolution of EN, which has also rarely been described elsewhere.^27^^,^^28^ The majority of patients in this cohort required hospitalization, including most patients with disseminated disease and nearly half of those with pneumonia. Patients with disseminated disease had prolonged hospitalizations averaging over 4 months and required increased outpatient support as they were on treatment for an average of 20 months longer (which does not even encompass the patients with CM on lifelong treatment). This underscores the substantial healthcare burden associated with severe coccidioidomycosis. Consistent with prior data, Black/African American children in our cohort were disproportionately affected by disseminated disease. Interestingly, immunocompromised status was not associated with increased risk of dissemination in our cohort; however, this may reflect the sample size being insufficient to detect a difference, early detection during pre-transplant screening^29^ and/or antifungal prophylaxis following transplantation in some patients.^17^

Using a recently published classification system,^18^ we observed that pediatric patients with dissemination often have musculoskeletal and CNS involvement. Our findings also align with previous data that *Coccidioides* CF titers >1:16 indicate an elevated risk of extrapulmonary dissemination,^9^^,^^17^ and this should warrant a careful history, physical exam, and additional workup to look for these manifestations accordingly. Almost one-third of the patients in our cohort underwent a surgical intervention; thus, pediatric infectious diseases physicians should anticipate the need for source control in select patients with pulmonary or musculoskeletal disease^30^ and support neurosurgical management of intracranial pressure for CNS disease.^22^

Adult series suggest that only one-third of patients with primary coccidioidomycosis require medical attention, and even fewer require treatment. In contrast, and consistent with prior pediatric data, we observed high treatment rates, with 90% of children initiated on fluconazole, even when uncomplicated coccidioidomycosis was diagnosed. The majority of patients seen at our center were referred from pediatricians or other hospitals in highly endemic counties, and high treatment rates may reflect reticence with observation alone in the pediatric population. Consistently high treatment rates, in prior case series and ours, make it difficult to determine the proportion of undiagnosed pediatric coccidioidomycosis that might otherwise self-resolve without antifungal therapy. It is also possible that the referral bias at our institution may contribute to the high percentage of patients receiving antifungal therapy, as children managed expectantly without treatment may remain under the care of their local pediatricians and thus not be captured in our cohort.

Fluconazole is the preferred, first-line therapy, with weight-based dosing (6-12 mg/kg) favored in younger children and a minimum of 400 mg daily being used in older children per guidelines, with increased dosing (800-1200 mg) in cases of severe disease.^17^ The therapy of one-third of our treated patients was ultimately changed to a mold-active azole as their final therapy, either due to adverse effects on fluconazole or concerns for treatment failure, including all but 1 patient with CM. This likely reflects our center receiving referrals for patients worsening on fluconazole but is consistent with high rates of apparent fluconazole failure previously reported by others, especially in CM.^11^^,^^26^ The fluconazole MICs in our cohort also trended higher in recent years, consistent with a recent large-scale in vitro evaluation demonstrating that over a third of recent *Coccidioides* isolates had MICs *>*16 mg/L.^13^ This suggests higher doses of fluconazole with therapeutic drug monitoring^31^ may be needed as a first-line agent or moving on to a mold-active azole for severe disease when higher MICs or clinical failure are seen. Itraconazole was rarely used in our cohort due to concerns about absorption, gastrointestinal side effects, and a frequent dosing schedule, especially in younger children. Mold-active triazoles were largely well tolerated, even when high doses were used to achieve desired trough levels (reflecting the complex pharmacokinetics and drug interactions these pose). However, the single case of voriconazole-induced fluorosis, a well-recognized toxicity, also underscores the importance of long-term safety monitoring.^32^^,^^33^ Dual therapy with a formulation of amphotericin B and an azole antifungal agent was used in patients with severe musculoskeletal disease^17^ or patients with meningitis with other sites of dissemination. Combination treatment with echinocandins has been described with success in prior case reports for salvage therapy but, without further data and increasing availability of novel azoles, is not currently recommended.^34^

Our study has limitations inherent to its single-center retrospective design, and findings may not be generalizable. Management strategies are also influenced by local expert opinions. Another limitation is a selection bias as our institution is a major referral center for patients in the highly endemic neighboring counties, artifactually enriching our cohort for disseminated disease. However, this is also a strength of this series as it offers valuable insight into the variable presentations and difficult management decisions for the most complex patients. There is also a small risk of misclassification despite manual chart review. Nonetheless, the strength of our dataset lies in its longitudinal scope, detailed clinical review, and reflection of contemporary management in an era of broader access to mold-active agents, intrathecal therapy, and immunomodulatory strategies.

In summary, pediatric coccidioidomycosis at our institution has become increasingly common and increasingly severe, with a substantial rise in disseminated disease in recent years. These trends emphasize the need for heightened clinical vigilance, early recognition of extrapulmonary disease, incorporation of a standardized classification system to guide evaluation, and proactive multidisciplinary management. As the endemic area expands, centers beyond the southwestern United States will likely encounter similar patients, and our experience may help inform treatment strategies and system-level preparedness for this emerging pediatric burden.

## Supplementary Material

patient-consent-form-TG_piag019
