# Supplementary material for: Rising burden of severe pediatric coccidioidomycosis: a 25-year single-center study
Source: J Pediatric Infect Dis Soc. 2026 Mar 12;15(4):piag019. doi: 10.1093/jpids/piag019 (PMC13131227; doi:10.1093/jpids/piag019)
Supplement: patient-consent-form-TG_piag019 [file patient-consent-form-tg_piag019.zip › KC Informed Consent.pdf]

CONSENT FORM FOR CASE REPORTS[1]

For a patient's consent to publication of information about them in a journal or thesis

Name of person described in article or shown in photograph: Kei'marie Callahan

Subject matter of photograph or article: Scalp infection

Title of article: TBD

Medical practitioner or corresponding author: Richard Sleightholm

I Terriva Porter [insert full name] give my consent for this information about MYSELF OR MY CHILD OR WARD/MY RELATIVE [insert full name]: Kei Marie Callahan, relating to the subject matter above ("the Information") to appear in a journal article, or to be used for the purpose of a thesis or presentation.

I understand the following:

1. The Information will be published without my name/child's name/relatives name attached and every attempt will be made to ensure anonymity. I understand, however, that complete anonymity cannot be guaranteed. It is possible that somebody somewhere - perhaps, for example, somebody who looked after me/my child/relative, if I was in hospital, or a relative - may identify me.
2. The Information may be published in a journal which is read worldwide or an online journal. Journals are aimed mainly at health care professionals but may be seen by many non-doctors, including journalists.
3. The Information may be placed on a website.
4. I can withdraw my consent at any time before online publication, but once the Information has been committed to publication it will not be possible to withdraw the consent.

Signed: [signature] Date: 4-3-24

Signature of requesting medical practitioner/health care worker:

[signature] Date: 4-3-24

---

[1] Adapted from *BMJ Case Reports* consent form.
Division Research Development and Support, Faculty of Health Sciences, Stellenbosch University, South Africa. Consent form for case reports. Version1. Sept 2008.
