# Supplementary material for: Rising burden of severe pediatric coccidioidomycosis: a 25-year single-center study
Source: J Pediatric Infect Dis Soc. 2026 Mar 12;15(4):piag019. doi: 10.1093/jpids/piag019 (PMC13131227; doi:10.1093/jpids/piag019)
Supplement: patient-consent-form-TG_piag019 [file patient-consent-form-tg_piag019.zip › Supplemental Table 1.docx]

**Supplemental table 1. Categorization of patients based on previously published classification schema to determine clinical variants (amended to remove death category)**

| **Clinical Features** | **Category**[**^a^**](https://pmc.ncbi.nlm.nih.gov/articles/PMC10753911/) | **Subcategory**[**^a^**](https://pmc.ncbi.nlm.nih.gov/articles/PMC10753911/) | **Details** | **Our Cohort** |
| --- | --- | --- | --- | --- |
| Unrecognized coccidioidomycosis in the past | 1 | … | No clinical recognition of coccidioidal disease but evidence of prior infection | **5% (4)** |
| Uncomplicated pulmonary coccidioidomycosis, No evidence of extrapulmonary dissemination | 2 | … | Proved or probable coccidioidal lung disease with or without antifungal therapy; may have chest radiographic evidence of effusion or parenchymal disease, lymphadenopathy | **58% (47)** |
| Complicated pulmonary coccidioidomycosis | 3 | 3A | Pulmonary disease with simple cavitation | **5% (4)** |
| No evidence of extrapulmonary dissemination |  | 3B | Fibrocavitary lung disease | **0** |
|  |  | 3C | Persistent pulmonary disease for >6 mo despite therapy | **1% (1)** |
|  |  | 3D | Respiratory failure with or without multisystem organ failure | **3% (2)** |
| Extrapulmonary dissemination of coccidioidomycosis without meningitis or other CNS involvement | 4 | 4A | Extrapulmonary dissemination without meningitis; skin only | **0** |
|  |  | 4B | Extrapulmonary dissemination without meningitis to locations other than skin (eg, osteomyelitis, infective arthritis, or visceral organs) | **12% (10)** |
| Coccidioidal meningitis (or other CNS involvement of coccidioidomycosis) | 5 | 5A | Meningitis; no other evidence of extrapulmonary dissemination | **5% (4)** |
|  |  | 5B | Meningitis with additional dissemination to skin (only) | **3% (2)** |
|  |  | 5C | Meningitis with extrapulmonary dissemination to skin and other organs | **9% (7)** |
