# Supplementary figures and images for: Rising burden of severe pediatric coccidioidomycosis: a 25-year single-center study
Source: J Pediatric Infect Dis Soc. 2026 Mar 12;15(4):piag019. doi: 10.1093/jpids/piag019 (PMC13131227; doi:10.1093/jpids/piag019)

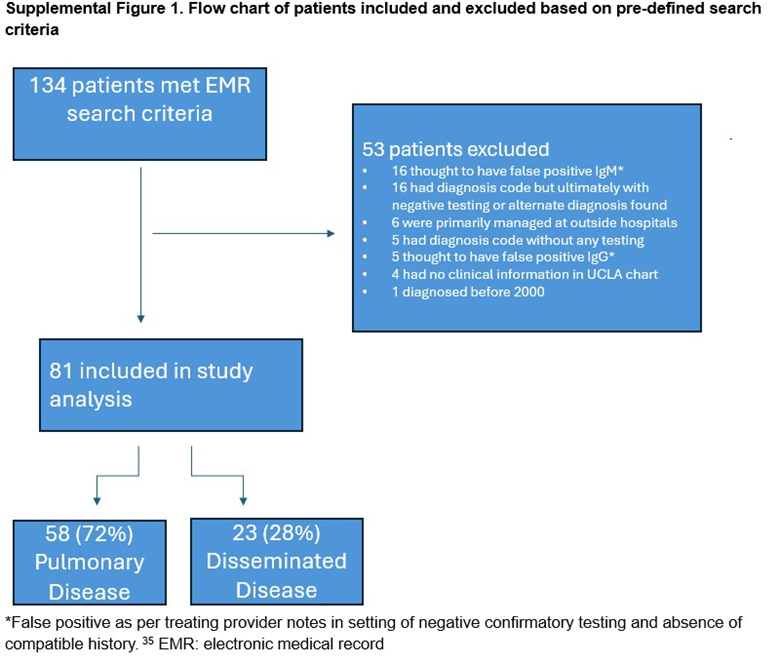

Supplement: patient-consent-form-TG_piag019 [file patient-consent-form-tg_piag019.zip › Supplemental_Figure_1.tif]
