# Supplementary material for: Rising burden of severe pediatric coccidioidomycosis: a 25-year single-center study
Source: J Pediatric Infect Dis Soc. 2026 Mar 12;15(4):piag019. doi: 10.1093/jpids/piag019 (PMC13131227; doi:10.1093/jpids/piag019)
Supplement: patient-consent-form-TG_piag019 [file patient-consent-form-tg_piag019.zip › patient-consent-form-AM.doc]

**CONSENT FORM FOR CASE REPORTS^[1]^**

**For a patient’s consent to publication of information about them in a journal or thesis**

Name of person described in article or shown in photograph: Abraham Martinez

Subject matter of photograph or article: Abraham Martinez

Title of article: Rising Burden of Severe Pediatric Coccidioidomycosis: A 25-Year Tertiary-Center Study

Medical practitioner or corresponding author: Sanchi Malhotra MD

I, Judy Rico, give my consent for this information about MY CHILD: Abraham Martinez, relating to the subject matter above (“the Information”) to appear in a journal article, or to be used for the purpose of a thesis or presentation.

I understand the following:

1. The Information will be published without my name/child’s name/relatives name attached and every attempt will be made to ensure anonymity. I understand, however, that complete anonymity cannot be guaranteed. It is possible that somebody somewhere - perhaps, for example, somebody who looked after me/my child/relative, if I was in hospital, or a relative - may identify me.
2. The Information may be published in a journal which is read worldwide or an online journal. Journals are aimed mainly at health care professionals but may be seen by many non-doctors, including journalists.
3. The Information may be placed on a website.
4. I can withdraw my consent at any time before online publication, but once the Information has been committed to publication it will not be possible to withdraw the consent.

Telephone consent obtained on: 12/18/2025 3:55 pm

Signature of requesting medical practitioner/health care worker:

 Date: 12/18/2025

1. Adapted from *BMJ Case Reports* consent form.
